# A lightweight multi-deep learning framework for accurate diabetic retinopathy detection and multi-level severity identification

**DOI:** 10.3389/fmed.2025.1551315

**Published:** 2025-04-02

**Authors:** Amad Zafar, Kwang Su Kim, Muhammad Umair Ali, Jong Hyuk Byun, Seong-Han Kim

**Affiliations:** ^1^Department of Artificial Intelligence and Robotics, Sejong University, Seoul, Republic of Korea; ^2^Department of Scientific Computing, Pukyong National University, Busan, Republic of Korea; ^3^Department of Mathematics, Institute of Mathematical Science, Pusan National University, Busan, Republic of Korea; ^4^Finance Fishery Manufacture Industrial Mathematics Center on Big Data, Pusan National University, Busan, Republic of Korea

**Keywords:** diabetic retinopathy, fundus imaging, deep learning model, lightweight model, transfer learning

## Abstract

Accurate and timely detection of diabetic retinopathy (DR) is crucial for managing its progression and improving patient outcomes. However, developing algorithms to analyze complex fundus images continues to be a major challenge. This work presents a lightweight deep-learning network developed for DR detection. The proposed framework consists of two stages. In the first step, the developed model is used to assess the presence of DR [i.e., healthy (no DR) or diseased (DR)]. The next step involves the use of transfer learning for further subclassification of DR severity (i.e., mild, moderate, severe DR, and proliferative DR). The designed model is reused for transfer learning, as correlated images facilitate further classification of DR severity. The online dataset is used to validate the proposed framework, and results show that the proposed model is lightweight and has comparatively low learnable parameters compared to others. The proposed two-stage framework enhances the classification performance, achieving a 99.06% classification rate for DR detection and an accuracy of 90.75% for DR severity identification for APTOS 2019 dataset.

## 1 Introduction

Diabetes is a growing global health concern, with approximately 830 million people affected worldwide in 2022, according to the World Health Organization (1). The prevalence of diabetes is expected to continue rising, resulting in an increasing number of people developing related complications, such as diabetic retinopathy (DR). Maintaining optimal visual health is essential for ensuring the well-being of individuals worldwide. Visual impairment and blindness pose significant threats to global health, driven by various factors including chronic diseases and unequal access to healthcare (2). As the number of people with DR grows from an estimated 103 million in 2020 to around 160 million by 2045, so does the number of individuals experiencing vision problems, posing a substantial challenge to public health and the economy (3). DR is a significant contributor to visual impairment and blindness worldwide. According to a study published by Al-Ghamdi (4), in Saudi Arabia, individuals over the age of 50 experience higher rates of blindness and visual impairment. For instance, among those aged 60 years and above, about 20% have blindness, while as many as 66.2% have visual impairment. Being a chronic and insidious disease, DR generally affects persons with diabetes, often developing insidiously without overt early symptoms. If left unattended, DR can result in irreversible blindness.

DR can be effectively managed through regular screening, which enables the condition to be detected early and treated. Advanced techniques promote the diagnosing and severity identification of tiny lesions at higher resolution imaging of the fundus. There are primarily two forms in which DR manifests itself: proliferative DR (PDR) and non-proliferative DR (NPDR) (5). NPDR can be classified into four different severity levels, ranging from mild to severe (6). Typical symptoms in DR include microaneurysms, retinal hemorrhage, and exudates. In the early development of DR, there are usually only slight manifestations of microaneurysms. With its progress, along with significant intraretinal hemorrhages and venous beading or microvascular abnormalities, complications can become more severe (7). The formation of new blood vessels defines PDR, often accompanied by vitreous or retina hemorrhage (8). Fundus imaging plays an imperative role in the diagnosis of DR (9), enabling ophthalmologists to analyze lesions, decide on the severity, and hence recommend treatment.

Grading DR from a fundus image is difficult even for expert ophthalmologists because diagnosis involves several lesions and often overlapped borders of those lesions, making perfect diagnosis time-consuming and unreliable. Hence, computer-aided systems are needed to grade DR. Various computer-aided systems have been presented in the literature to grade the DR severity (10–14). Such solutions intend to assist ophthalmologists in correctly assessing fundus images to detect lesions.

Various image processing methods are used to automatically detect hemorrhages, one common retinal manifestation related to diabetic patients (10). Other works have focused on developing strategies for detecting microaneurysms (11), one of the primary indicators of DR. Dimensionality reduction using techniques such as principal component analysis has been used to enhance microaneurysm detection. Fuzzy C-means clustering has also been utilized to diagnose DR and maculopathy automatically (15). Other researchers have also tried curvelet-based edge enhancement and wideband bandpass filters to improve the contrast between lesions and retina background (16).

Several classical machine learning methods have been employed by researchers using decision trees, support vector machines (17), random forests (18), logistic regression (19, 20), and Gaussian naive Bayes (19) for DR grading. These algorithms extract features from images with the aid of image processing techniques. For instance, Lachure et al. (17) used erosion, dilatation, opening, and closure for feature detection by applying morphology-based image processing methods to differentiate microaneurysms from exudates. Recently, studies of deep features for local and global feature extractions from fundus images have been conducted (18). These features are then utilized to train classifiers and predict the final DR classification. Most of the classical machine-learning methods depend heavily on feature extraction, which is often a complicated and time-consuming process. This may not capture all the intricate characteristics necessary for accurate classification.

In contrast, deep learning techniques have emerged as a powerful tool in imaging applications. Deep learning models have demonstrated remarkable success in identifying DR by automatically extracting complex features using convolutional layers (21, 22). This approach eliminates manual feature extraction, allowing for more efficient and accurate classification (12–14). Zhou et al. (23) introduced a multitasking deep neural network architecture for DR classification. By leveraging a multitasking approach, they forecasted labels through classification and regression, achieving an 84% kappa score. The interconnectedness of DR phases contributed to the achieved performance. In addition to deep learning models, transfer learning has also been explored for detection. Researchers have leveraged pre-trained models and fine-tuned them for DR detection, achieving promising results (24, 25). Furthermore, various feature selection approaches, including wrapper-based (26, 27) and filter-based methods (28), have been investigated for DR detection. These techniques aim to identify the relevant features, reducing the data dimensionality and improving model performance. Deep neural networks dominate image processing and computer vision applications. However, traditional deep learning models may not effectively capture complex, irregular objects. Despite advancements, multi-class categorization remains an area for improvement. On the other hand, transfer learning models are effective, but can be even more beneficial when fine-tuned on weights frozen with images that are highly correlated with the target task. This study aimed to enhance classification performance while minimizing layers and parameters.

This research presents a novel deep-learning architecture and framework for detecting DR and identifying its severity. The approach involves a two-stage process. Initially, a custom-designed deep learning lightweight classification model is developed to distinguish between healthy (no DR) and DR-affected (mild DR, moderate DR, severe DR, and PDR) fundus images. Upon identifying the presence of DR, the pre-trained model is reused using transfer learning to subclassify the severity of DR into specific categories, including mild, moderate, severe DR, and PDR. Leveraging the initial model’s frozen weights on related images facilitates the severity identification process. The proposed deep learning model and framework are validated using a publicly available online dataset. The proposed framework is compared to existing state-of-the-art models to check its simplicity and efficiency.

## 2 Materials and methods

This section provides a complete description of the methodologies and materials in this work, particularly the developed deep learning model and its framework. Additionally, it describes the data augmentation techniques used to tackle the dataset’s imbalanced issues.

### 2.1 Proposed DR detection multi-level severity identification framework

**Figure 1:**
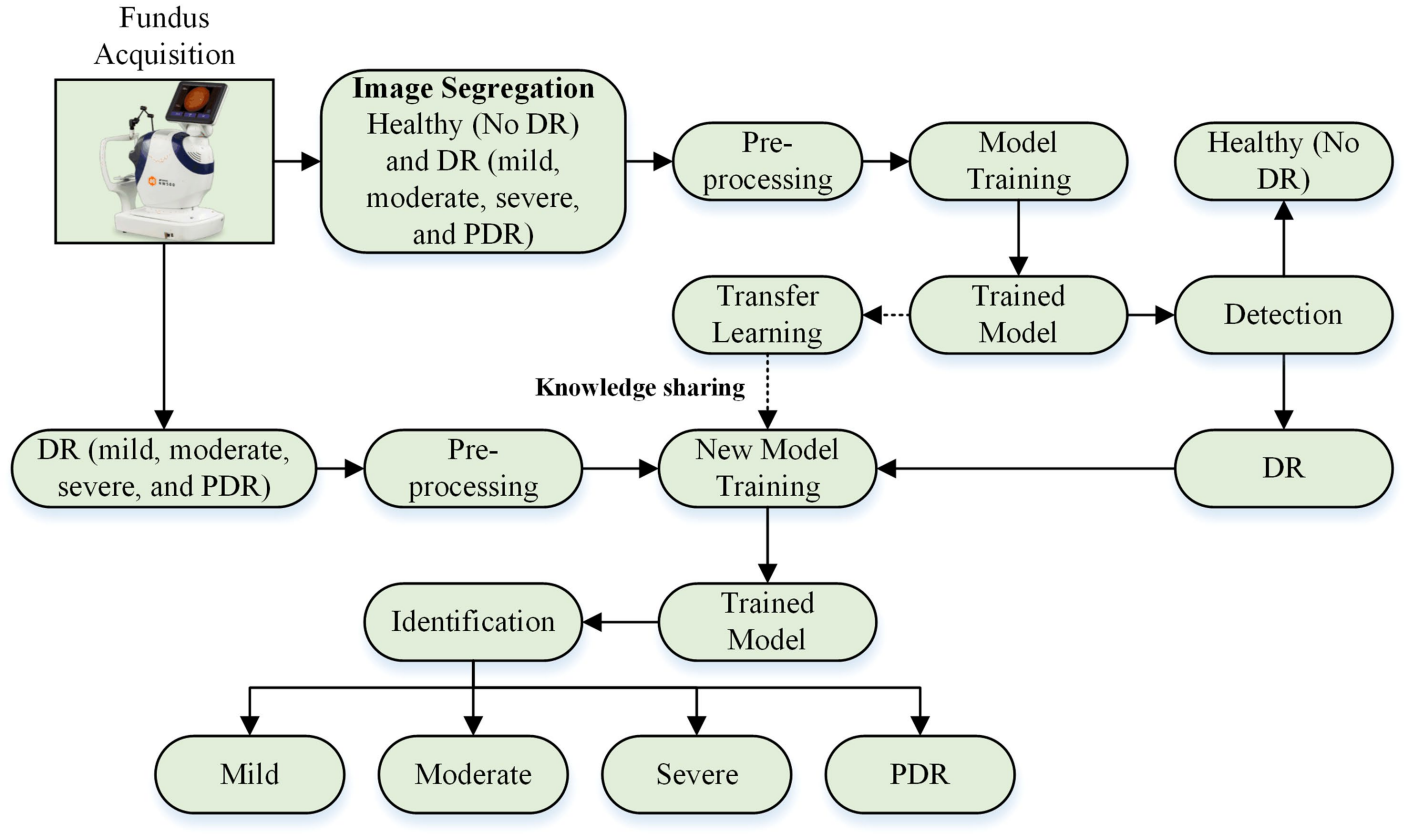
DR detection and severity identification framework using deep learning approach.

This research presents a deep learning-based approach for detecting and identifying the severity of DR. This approach is divided into two phases. Phase I involves designing a deep learning model to classify fundus images as healthy (no DR) and DR-affected (mild DR, moderate DR, severe DR, and PDR). Stage II utilizes the trained model from Stage I to further categorize DR retinas into mild, moderate, severe DR, and PDR. The overall framework is illustrated in Figure 1.

### 2.2 Proposed deep learning model and transfer learning model for DR detection and severity identification

This section explores two deep learning approaches for DR detection and severity identification: (i) isolated deep learning networks and (ii) transfer learning.

An isolated model is trained from scratch without prior knowledge (29), whereas transfer learning leverages pre-acquired knowledge from other models (30). The transfer learning approach involves training a base model on a base dataset and then reusing the learned features to train a new model for a specific task (31–33). This study employs a developed deep learning model approach for transfer learning, where a pre-trained model is reutilized by modifying its architecture to accommodate the specific task of DR severity identification.

Two deep learning models are examined: the isolated deep learning model and the transfer learning-based deep learning model. The isolated deep learning model is designed to categorize fundus images into healthy (no DR) and DR-affected (mild DR, moderate DR, severe DR, and PDR) classes, while the transfer learning-based models are used to subclassify DR-affected retinas into mild, moderate, severe DR, and PDR.

**Figure 2:**
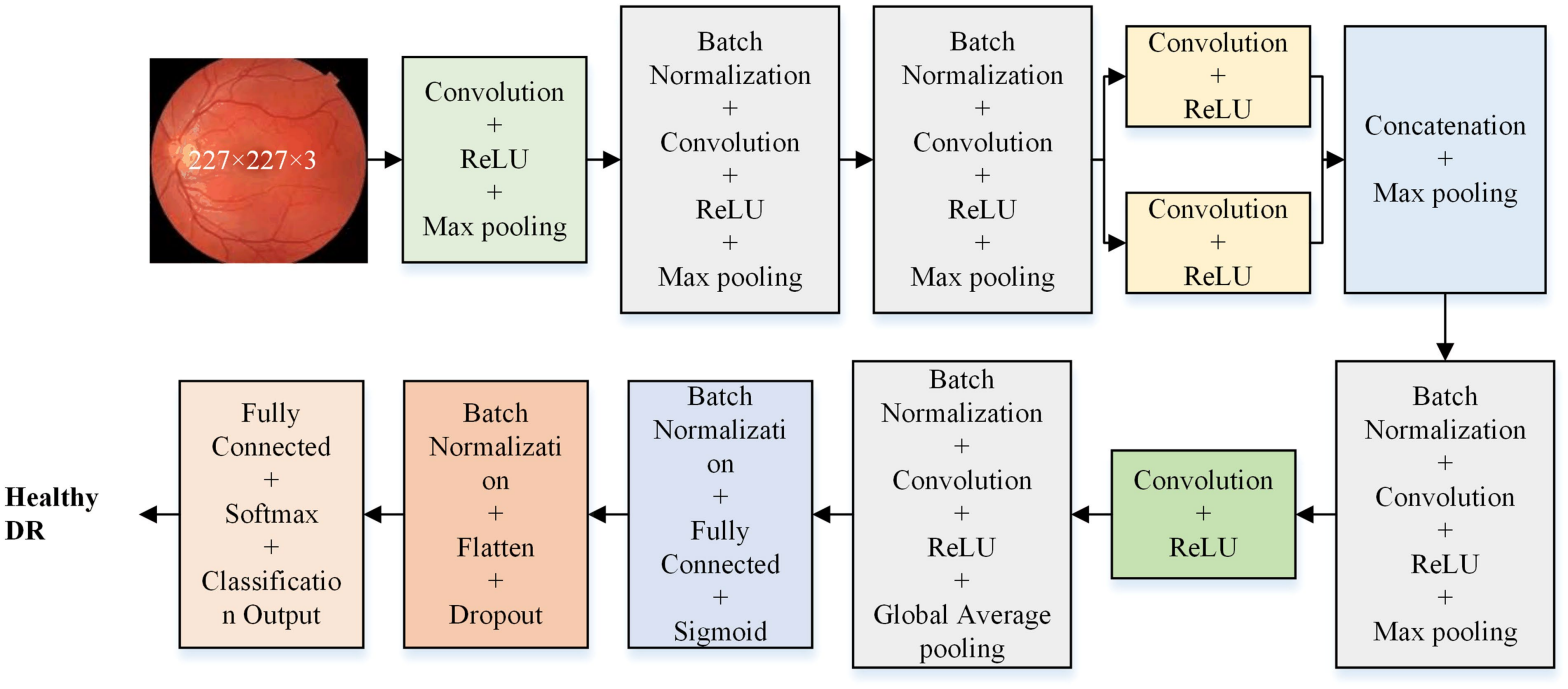
Proposed deep learning model architecture for DR detection.

The isolated model consists of five primary layers: input, convolution, pooling, fully connected, and classification. The features are extracted in convolution and pooling layers, while the fully connected and classification layers facilitate prediction. This study develops and evaluates various isolated models with different architectures (36-layer, 33-layer with one parallel branch, 37-layer with one parallel branch, 41-layer with one parallel branch, and 42-layer with two parallel branch) to determine the optimal model for DR detection. Figure 2 illustrates the detailed architecture of the optimal model (37-layer with one parallel branch) used for further analysis of DR detection.

**Figure 3:**
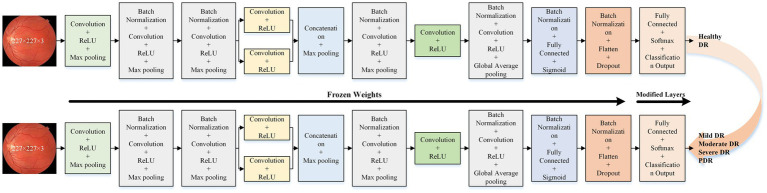
Proposed transfer learning deep learning model architecture for DR severity identification.

The optimal isolated deep learning model is reused for DR severity identification. The last three layers of the pre-trained model are replaced and retrained to accommodate the multi-class classification task. Figure 3 presents the detailed architecture of the transfer learning model used for DR severity identification.

### 2.3 Dataset and preprocessing

**Figure 4:**
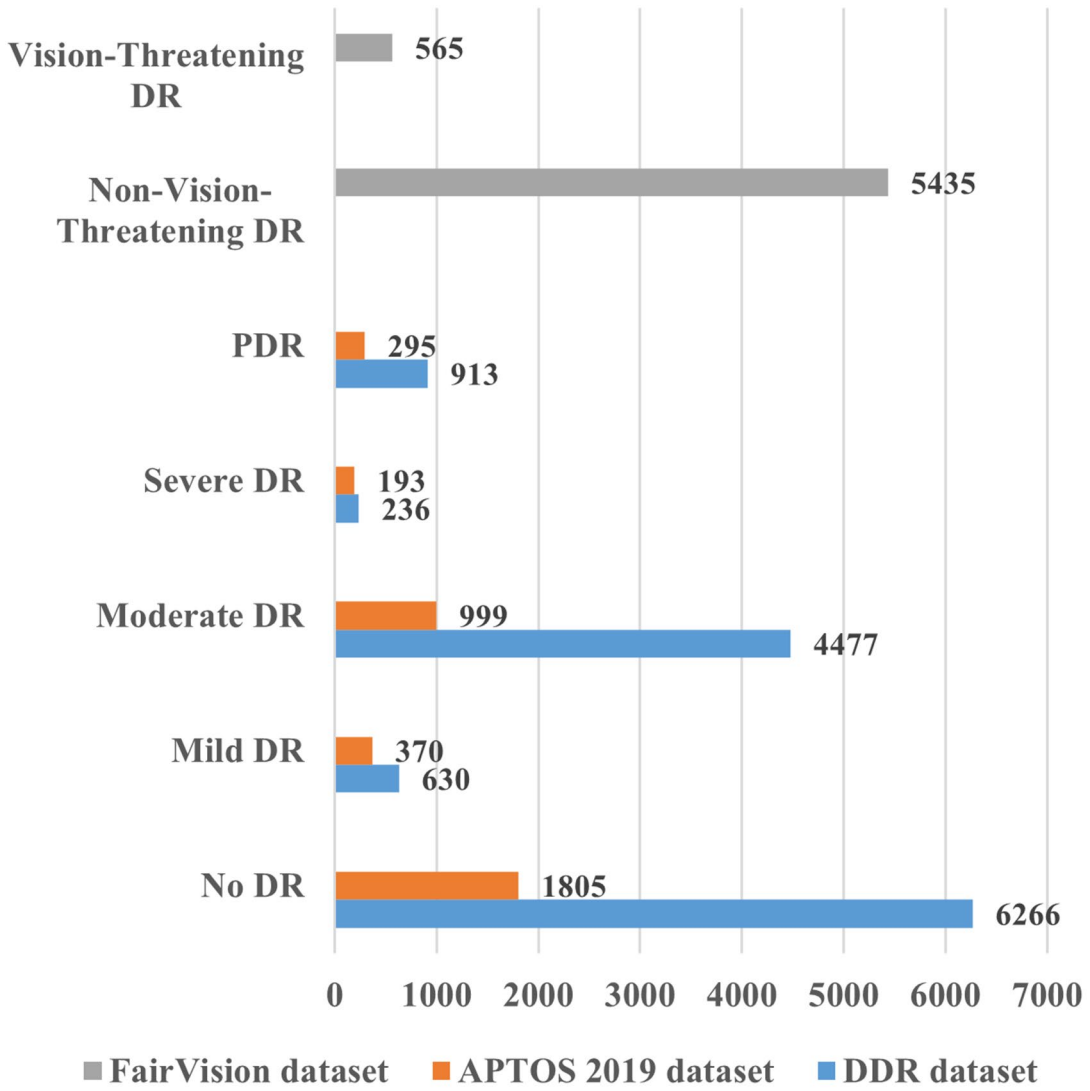
Overview of the various datasets for DR detection and severity identification.

The study employed the FairVision dataset (34), the Asia Pacific Tele-Ophthalmology Society (APTOS) 2019 dataset (https://www.kaggle.com/c/aptos2019-blindness-detection/overview, accessed on 20 November 2024), and the DDR dataset (https://www.kaggle.com/datasets/mariaherrerot/ddrdataset, accessed on 28 February 2025). The dataset comprises fundus photographs captured from patients, representing a diverse range of imaging conditions. The FairVision, APTOS 2019, and DDR datasets included 6,000, 3,662, and 12,522 fundus images, respectively. Figure 4 offers more information regarding the distribution of fundus images.

The developed model required uniform input images, which were achieved by cropping all images to a fixed size of 227 × 227 pixels. Normalization was performed using a zero-center approach during the preprocessing stage.

A significant challenge in deep learning is the scarcity of data. Increasing the dataset size enables models to learn more robust features and reduces overfitting risks. Our datasets were initially imbalanced, with varying image counts across classes. We employed data augmentation techniques to address this, including flipping, rotation, scaling, and translation. These augmentations enhanced the dataset’s size and image quality; details are presented in Table 1.

**Table 1:** Details about the images used for DR detection and severity identification.

Dataset	Category	Subcategory	Original images	Augmented images for DR detection	Augmented images for DR severity identification
FairVision Dataset	Non-vision-threatening DR	—	5,435	6,000	—
	Vision-threatening DR	—	565	6,000	—
APTOS 2019	Healthy (no DR)	Healthy (no DR)	1,805	4,000	—
	DR-affected	Mild DR	370	4,000	1,000
		Moderate DR	999		1,000
		Severe DR	193		1,000
		PDR	295		1,000
DDR Dataset	Healthy (no DR)	Healthy (no DR)	6,266	6,000	—
	DR-affected	Mild DR	630	6,000	1,500
		Moderate DR	4,477		1,500
		Severe DR	236		1,500
		PDR	913		1,500

## 3 Results

This research used MATLAB 2023a for all simulations and analyses on a personal computer, which has the following specs: Core i7, 12th Generation, 32 GB RAM, NVIDIA GeForce RTX 3050, 1 TB SSD, and 64-bit Windows 11 operating system. Using hold-out validation, we divided the dataset randomly into 80/20 ratios for model training and testing. The images used for model testing were not part of the training set. The initial parameters were as follows: 100 epochs, 0.9 momentum, 128 mini batch-size, and 0.001 learning rate. The stochastic gradient descent with momentum (SGDM) solver is used for training and testing.

The APTOS 2019 dataset was initially utilized to evaluate the proposed model’s performance. A preliminary analysis was conducted to identify the optimal layer configuration for the lightweight deep learning model. The results of this analysis are summarized in Table 2. Based on the findings, the 37-layer model was selected, as it demonstrated superior accuracy.

**Table 2:** Results of the ablation study performed for the selection of layers using APTOS 2019 dataset.

Developed models	Training loss	Training accuracy (%)	Validation loss	Validation accuracy (%)	Training time
33-layer (1 parallel branch)	8.33 × 10^−02^	100	1.0811	78.04	20 min 16 s
36-layer (no parallel branch)	1.04 × 10^−01^	100	1.2028	75.03	12 min 11 s
37-layer (1 parallel branch)	1.8 × 10^−02^	100	0.9649	80.08	14 min 28 s
41-layer (1 parallel branch)	5.41 × 10^−02^	100	1.2432	78.04	12 min 38 s
42-layer (2 parallel branch)	3.66 × 10^−02^	100	0.9579	77.63	15 min 56 s

Several state-of-the-art models, including VGG-16, MobileNet-v2, Efficient-b0, Inception-v3, ResNET-50, and GoogLeNet, were also trained and tested to compare comprehensively using APTOS 2019 dataset. The results of this evaluation are summarized in Table 3.

**Table 3:** Performance of various deep learning models against APTOS 2019 dataset.

Network	True class	Predicted class					Accuracy (%)	Cohen’s kappa	Training time	Learnable (M)
		Mild DR	Moderate DR	No DR	PDR	Severe DR				
VGG-16	Mild DR	42	19	8	4	1	76.13	0.64	12 min 49 s	134.2
	Moderate DR	29	137	8	17	9				
	No DR	3	1	357	0	0				
	PDR	12	25	4	13	5				
	Severe DR	2	16	0	12	9				
MobileNet-v2	Mild DR	37	25	9	2	1	79.9	0.69	51 min 40 s	2.2
	Moderate DR	8	153	11	8	20				
	No DR	4	3	354	0	0				
	PDR	3	22	0	24	10				
	Severe DR	0	19	0	2	18				
Efficient-b0	Mild DR	47	17	5	5	0	82.40	0.73	162 min 6 s	4
	Moderate DR	18	155	4	15	8				
	No DR	5	1	355	0	0				
	PDR	2	16	0	33	8				
	Severe DR	0	17	0	8	14				
Inception-v3	Mild DR	47	18	4	4	1	81.86	0.72	110 min 17 s	21.8
	Moderate DR	13	157	2	19	9				
	No DR	5	0	356	0	0				
	PDR	5	22	1	28	3				
	Severe DR	0	23	1	3	12				
ResNET-50	Mild DR	41	19	12	1	1	75.30	0.62	75 min 49 s	23.5
	Moderate DR	40	137	13	4	6				
	No DR	5	1	355	0	0				
	PDR	7	26	6	14	6				
	Severe DR	3	28	1	2	5				
GoogLeNet	Mild DR	50	19	4	1	0	80.8	0.71	25 min 27 s	5.9
	Moderate DR	21	156	3	10	10				
	No DR	8	1	351	1	0				
	PDR	5	28	0	21	5				
	Severe DR	1	19	0	5	14				
Proposed	Mild DR	36	33	1	2	2	80.08	0.70	14 min 28 s	1.3
	Moderate DR	24	146	4	17	9				
	No DR	6	2	353	0	0				
	PDR	6	17	2	32	2				
	Severe DR	4	11	0	4	20				

After comprehensively analyzing Table 3, it can be observed that the Efficient-b0 showed the highest classification rate of 82.4% (kappa value = 0.73), with almost 162 min 6 s training time, and also has 4 million learnable parameters. The second best is the Inception-v3 model with 81.86% classification accuracy (kappa value = 0.72), 110 min 17 s training time, and 21.8 million learnable parameters. ResNET-50 showed the lowest performance, with only 75.3% accuracy (kappa value = 0.62) and 23.5 million learnable parameters. Notably, our proposed deep learning model demonstrated a reasonable classification rate of 80.08% (kappa value = 0.70), with a significantly shorter training time of just 14 min 28 s. As shown in Table 3, the model correctly classifies 587 out of 733 fundus images. Moreover, the developed network has the fewest learnable parameters compared to the other.

**Figure 5:**
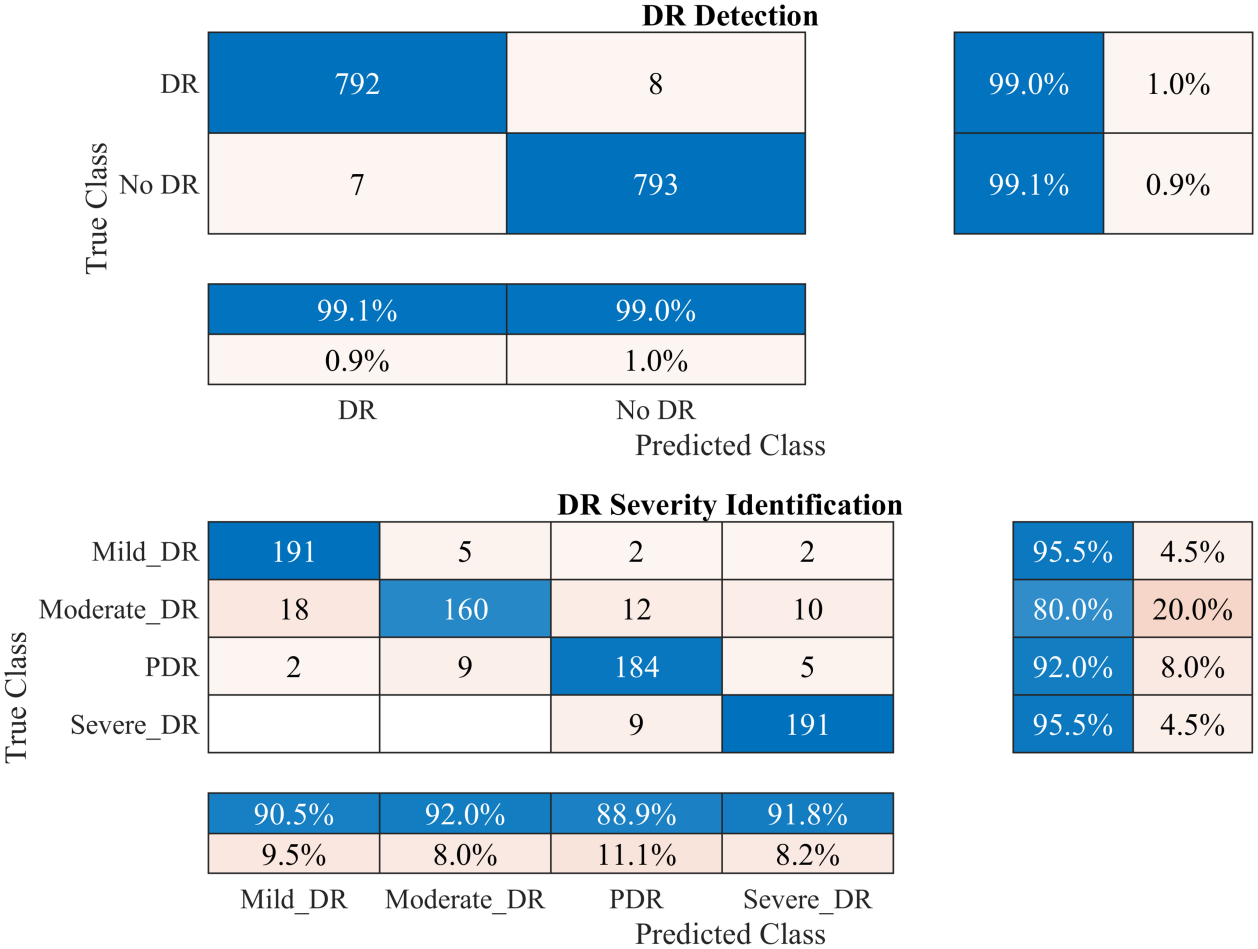
Performance of the developed deep learning model and DR detection and severity identification framework for the APTOS 2019 dataset.

**Figure 6:**
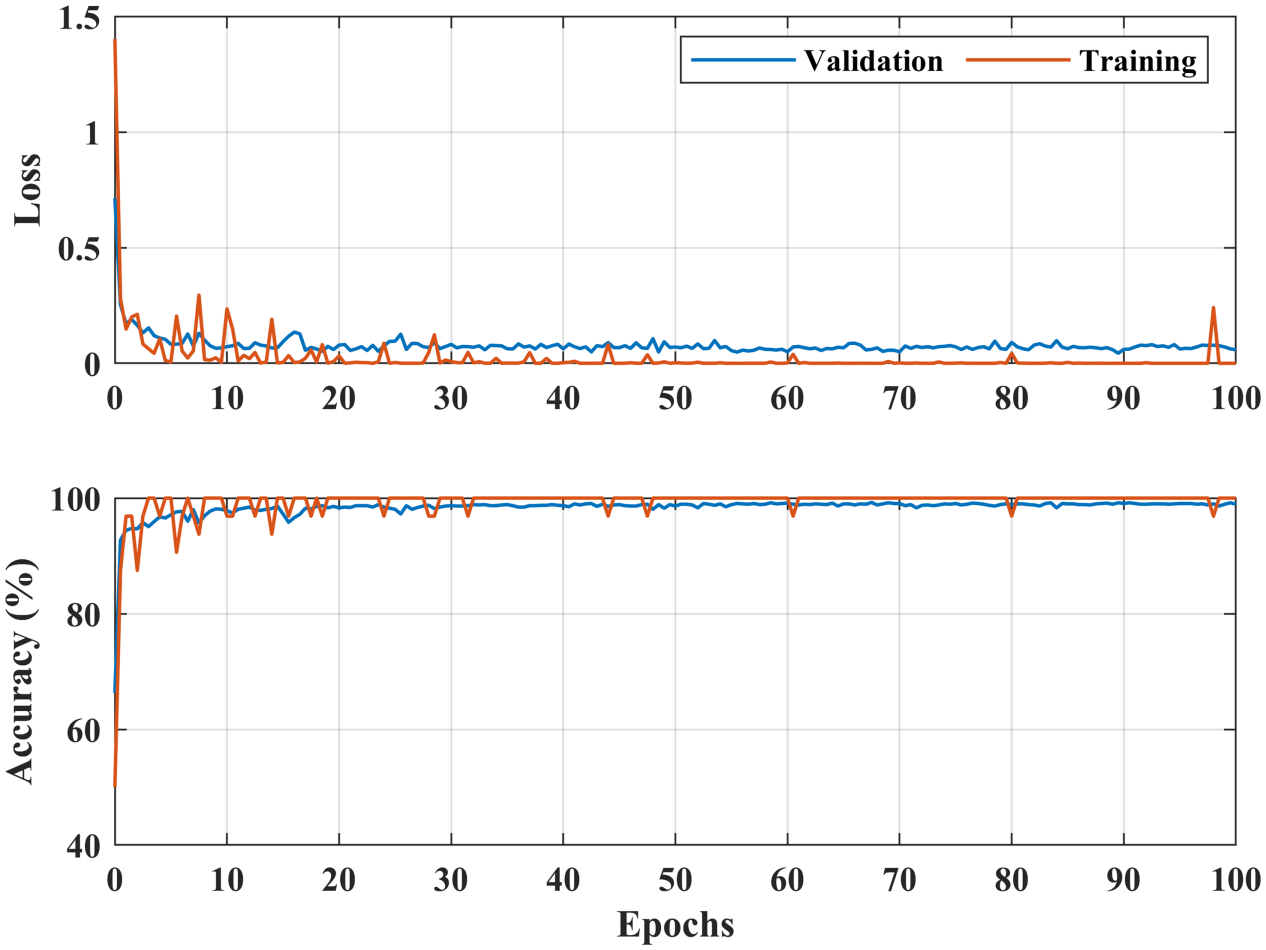
Learning curves DR detection for the APTOS 2019 dataset.

**Figure 7:**
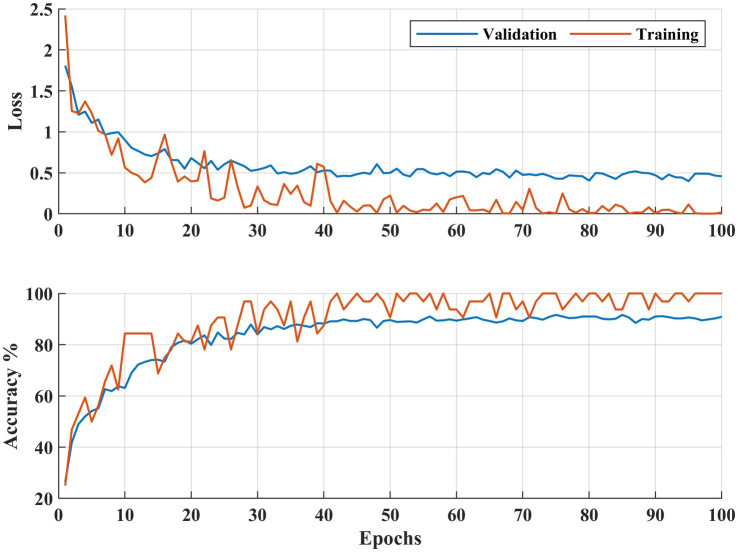
Learning curves DR severity identification for the APTOS 2019 dataset.

Therefore, the proposed model was employed to investigate DR detection and severity identification using the APTOS 2019 dataset, as discussed in section 2. The process involves initially distinguishing between healthy and DR-infected fundus images and detecting the DR severity in the second stage. Data augmentation was applied to address dataset imbalance, as illustrated in Table 1. The outcomes of this analysis for the APTOS 2019 dataset are presented in Figures 5–7.

Figure 5 demonstrates that the presented methodology effectively enhances the accuracy of DR detection. Specifically, the model achieved a high accuracy rate of 99.06% (kappa value = 0.98), correctly classifying 792 out of 800 DR images and misclassifying only seven healthy (no DR) fundus images.

In the case of DR severity level identification, some misclassification occurred, with 40 out of 200 fundus images for moderate DR class. However, these numbers are relatively low compared to the true positives. The model performed strongly in identifying mild and severe DR classes, with 95.5% recall for both classes (see Figure 5). Overall, the approach accomplished a high classification rate of 90.75% (kappa value = 0.88) for DR severity identification.

**Figure 8:**
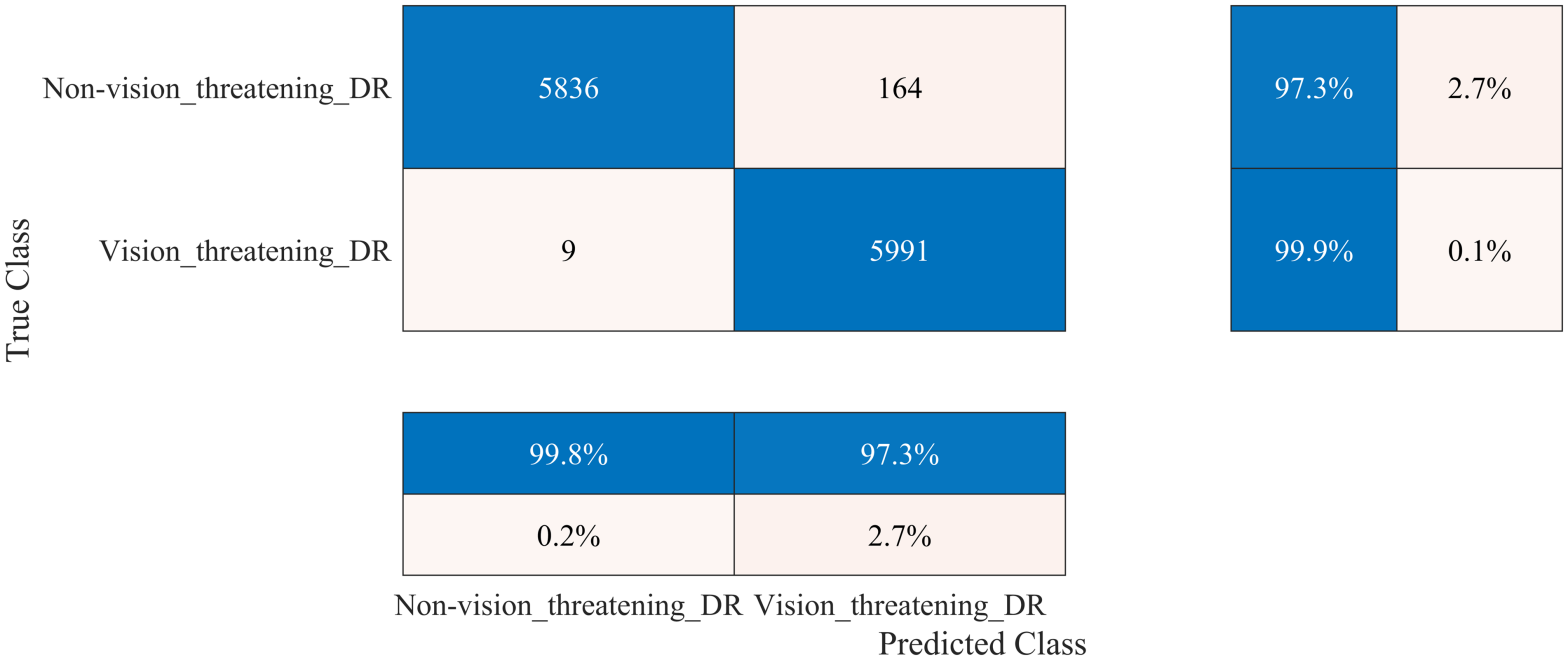
Performance of the developed deep learning model and DR detection framework for the FairVision dataset.

**Figure 9:**
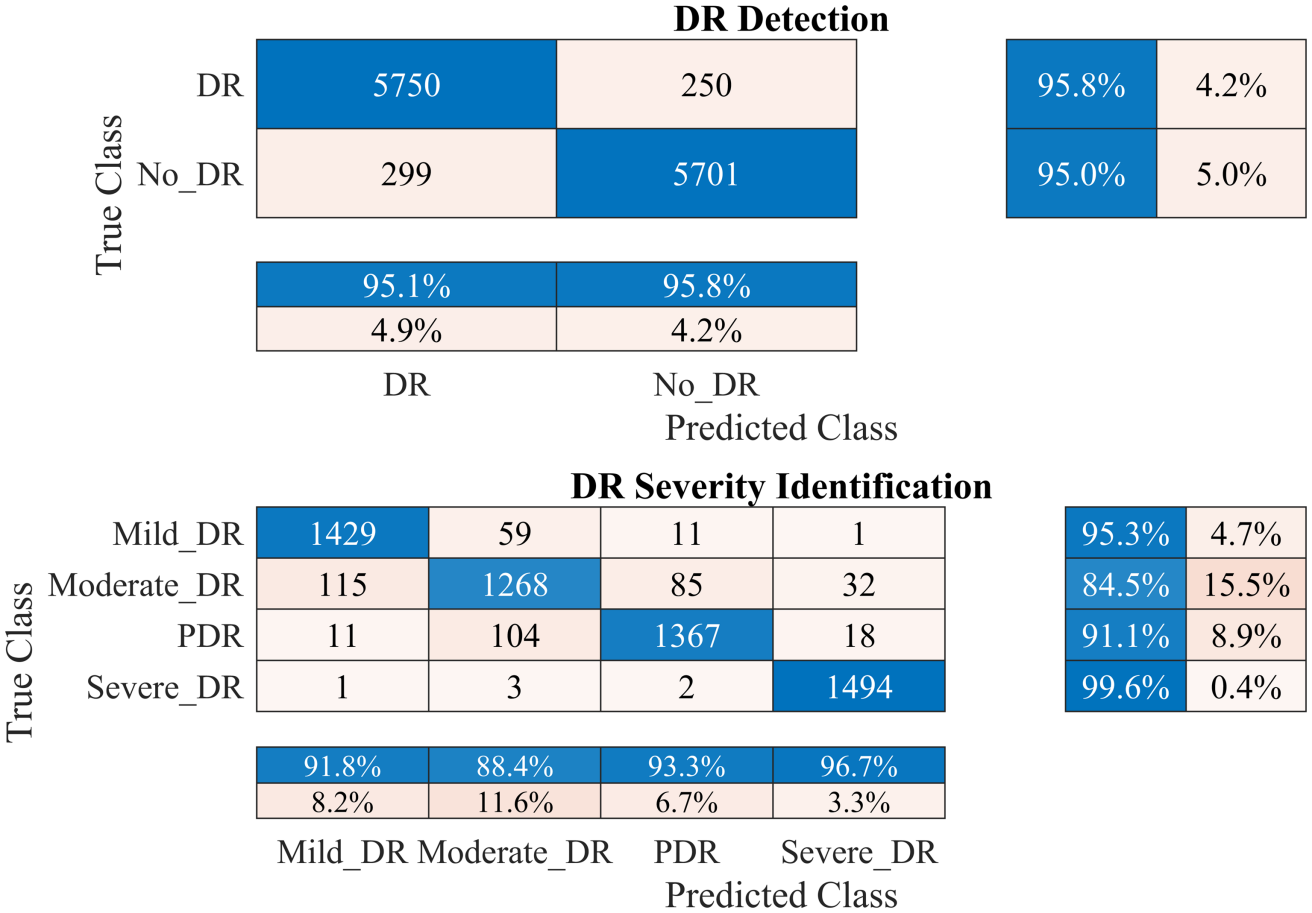
Performance of the developed deep learning model and DR detection and severity identification framework for the DDR dataset.

Figures 6, 7 illustrate both trained models’ learning curves, showing that they become stable after approximately 40 epochs. To further validate the generalizability of the proposed approach, we evaluated its performance on two additional datasets: FairVision and DDR (dataset details are provided in Figure 4 and Table 1). The results obtained from these datasets using 5-fold cross-validation are presented in Figures 8, 9.

The FairVision dataset offered two classes for DR detection: non-vision-threatening DR and vision-threatening DR. However, the dataset was highly imbalanced, comprising 5,435 images of non-vision-threatening DR and 565 images of vision-threatening DR. To address this imbalance, we applied an augmentation approach, resulting in 6,000 images for each class, as shown in Table 1. Our model achieved a high classification rate of 98.56% (kappa value = 0.97) to distinguish between non-vision-threatening DR and vision-threatening DR class, as shown in Figure 8. Similarly, the DDR dataset is also highly imbalanced, comprising 6,266, 630, 4,477, 236, and 913 images of none, mild, moderate, severe, and PDR. We applied an augmentation approach to address this imbalance, resulting in 6,000 images for the no DR class and 1,500 images for the remaining DR classes (1,500 × 4 = 6,000). The model again achieved a high classification rate of 95.43% (kappa value = 0.91) for detecting DR and 92.63% (kappa value = 0.90) for DR severity identification, as shown in Figure 9. The results of these well-known mainstream public datasets further validated the performance of the proposed model and multistage approach. Furthermore, a comparison of the proposed approach with other studies is presented in Table 4.

**Table 4:** Comparative analysis of classification performance with state-of-the-art models on the APTOS 2019 dataset.

Study	Results (%)
Kumar et al. (39)	75.50
Dondeti et al. (40)	77.9
Bodapati et al. (41)	97.41 (for DR detection) 80.96 (for severity identification)
Gangwar and Ravi (42)	82.18
Bodapati et al. (43)	82.54
Kassani et al. (38)	83.09
Bodapati et al. (24)	84.31
Shaik and Cherukuri (37)	90.45 (for DR detection) 84.31 (for severity identification)
Yang et al. (36)	85.60
Alam et al. (35)	87
Proposed	99.06 (for DR detection) 90.75 (for severity identification)

## 4 Discussion

DR is one of the major vision-threatening conditions faced by the healthcare department, which is quite challenging to diagnose and manage. Grading the severity of DR is vital for timely management and prevention of vision loss. This study presents a novel deep-learning network and framework for determining the detection and severity of DR, which would help the healthcare professional community manage patients effectively and efficiently.

An ablation study was conducted, where various deep-learning models were designed and tested for DR detection and severity identification. The 37-layer deep-learning model with a single parallel branch was selected due to its high classification accuracy and low training time (Table 2). Subsequently, various pre-trained models were trained and tested alongside the proposed 37-layer model, with detailed results presented in Table 3. After thorough analysis, it was observed that the developed deep-learning model achieved reasonable accuracy with reduced training time and minimal learnable parameters (1.3 million), making it lightweight. Onward, the model was initially trained to detect DR using augmented fundus images (APTOS 2019), and the results are presented in Figure 5. The model demonstrated a high performance for DR detection (99.06%). Furthermore, in the case of DR severity detection, the results achieved a high severity level detection accuracy of 90.75%. To validate the proposed model and approach further, it was evaluated using the FairVision and DDR datasets. The results demonstrated excellent performance, with a classification accuracy of 98.56% on the FairVision dataset for distinguishing between non-vision-threatening and vision-threatening DR (see Figure 8). On the DDR dataset, our model achieved an accuracy of 95.43% for DR detection and 92.63% for severity level identification (see Figure 9).

APTOS 2019 is a benchmark dataset used for the performance evaluation of many different models in detecting DR severity. Table 4 summarizes the performance of state-of-the-art models using this dataset. Recently, Alam et al. (35) investigated the use of the swapping assignments across multiple views technique for DR grading in conjunction with contrasting cluster assignments in retinal fundus images. The model achieved an accuracy of 87% with fewer parameters and layers. In another recent study (36), the authors introduced a novel transformer-based model called TMILv4 with an accuracy of 85.6%. However, its sensitivity was relatively low, at 73.7%. Similarly, Shaik and Cherukuri (37) combined a convolutional autoencoder with a neural support vector machine. They reported an accuracy of 84.31%, although the sensitivity score was as low as 66.16%. Contrarily, Kassani et al. (38) used the modified Xception network; it had higher sensitivity but lower accuracy at 83.09%. In contrast, the proposed model beats those state-of-the-art deep learning and transformer-based models, as represented in Table 4.

## 5 Conclusion

This study presents a novel, lightweight, deep-learning DR detection and severity identification framework. A 37-layer deep-learning model is designed to detect the DR using fundus images. The model is validated using a publicly available dataset and augmented using image augmentation techniques to balance classes. Comprehensive experiments are conducted to evaluate the proposed model’s performance using various metrics. The results demonstrate exceptional accuracy, with the proposed model achieving 99.06% accuracy for DR detection. Notably, the model has significantly fewer learnable parameters than existing models. The trained model is reutilized to evaluate its performance in detecting DR severity levels using transfer learning, yielding a high classification accuracy of 90.75%. Furthermore, our proposed model achieved high accuracy rates of 98.56% for FairVision, and 95.43% in detecting DR and 92.63% its severity for DDR dataset. The outstanding performance surpasses previous deep learning algorithms, providing superior DR detection and severity identification results. This work serves as a reference for classifying fundus images using deep learning techniques, offering a lightweight, efficient, and robust model.

## Data Availability

The original contributions presented in the study are included in the article/supplementary material, further inquiries can be directed to the corresponding author.
